# International shipping as a potent vector for spreading marine parasites

**DOI:** 10.1111/ddi.13592

**Published:** 2022-07-02

**Authors:** Katrina M. Pagenkopp Lohan, John A. Darling, Gregory M. Ruiz

**Affiliations:** 1 Coastal Disease Ecology Laboratory, Smithsonian Environmental Research Center, Edgewater, Maryland, USA; 2 Center for Environmental Measurement and Modeling, United States Environmental Protection Agency, Durham, North Carolina, USA; 3 Marine Invasions Research Laboratory, Smithsonian Environmental Research Center, Edgewater, Maryland, USA

**Keywords:** ballast water, dispersal, estuarine, marine disease, metabarcode, metazoan parasite, non-indigenous, non-native, phytoplankton, protistan parasite, zooplankton

## Abstract

**Aim:**

The global shipping fleet, the primary means of transporting goods among countries, also serves as a major dispersal mechanism for marine invasive species. To date, researchers have primarily focussed on the role of ships in transferring marine macrofauna, often overlooking transfers of associated parasites, which can have larger impacts on naïve host individuals and populations. Here, we re-examine three previously published metabarcode datasets targeting zooplankton and protists in ships’ ballast water to assess the diversity of parasites across life stages arriving to three major US ports.

**Location:**

Port of Hampton Roads in the Chesapeake Bay, Virginia; Ports of Texas City, Houston and Bayport in Galveston Bay, Texas; and Port of Valdez in Prince William Sound, Alaska.

**Methods:**

We selected all known parasitic taxa, using sequences generated from the small subunit gene (SSU) from ribosomal RNA (rRNA) amplified from (1) zooplankton collected from plankton tows (35 and 80 μm datasets) and (2) eukaryotes collected from samples of ships’ ballast water (3 μm dataset).

**Results:**

In all three datasets, we found a broad range of parasitic taxa, including many protistan and metazoan parasites, that infect a wide range of hosts, from teleost fish to dinoflagellates. Parasite richness was highest in the 3 μm dataset and relatively uniform across arrival regions. Several parasite taxa were found in high relative abundance (based on number of sequences recovered) either in ships entering a single or across multiple regions.

**Main Conclusions:**

The ubiquity, diversity and relative abundance of parasites detected demonstrate ships are a potent vector for spreading marine parasites across the world’s oceans, potentially contributing to reported increases in outbreaks of marine diseases. Future research is urgently needed to evaluate the fate of parasites upon arrival and the efficacy of ballast water treatment systems to reduce future transfers and colonization.

## 1 | INTRODUCTION

Parasites play many important roles in ecosystems, influencing food web dynamics (Lafferty et al., 2006) and modulating predator–prey interactions and inter and intra- specific competition within communities (Hatcher et al., 2006). Parasites can also cause infectious diseases that result in widespread community and ecosystem-level changes in the ocean (Harvell et al., 2004), with research suggesting these infectious diseases may be increasing over time (Tracy et al., 2019; Ward & Lafferty, 2004). Outbreaks occur for multiple reasons including changes in environmental conditions altering host–parasite interactions (Harvell et al., 2004; Hewson et al., 2014) and the introduction of non-native parasites to a new (previously uncolonized) region, potentially causing disease in naïve hosts (Dunn & Hatcher, 2015).

In coastal waters, aquatic non-native species are primarily introduced through human-assisted dispersal via maritime traffic, including the diverse biota associated with either the ballast water discharged by vessels or the biofouling organisms on ships’ hulls (Bailey et al., 2020). The global shipping industry has increased its shipping volume worldwide resulting from an increased demand in international trade markets. Although many studies have shown that the detection rate of marine invasions is increasing (Ruiz et al., 2015), few studies have included parasites in calculations of the number of marine invasive species (Poulin, 2017; but see Blakeslee et al., 2013; Torchin et al., 2002, 2003; Torchin & Mitchell, 2004).

Ships’ ballast water is recognized generally as an important potential source of parasites and pathogens in the marine environment (Harvell et al., 2004), but few studies have examined ballast water or hull-fouling communities to characterize the associated microbes or parasites. Past studies that focussed on microbial taxa in ballast tanks generally found a high diversity present (Drake et al., 2001, 2007; Ruiz et al., 2000), particularly when utilizing new genetic methods (e.g., metabarcoding) for examining these communities (Kim et al., 2015; Lymperopoulou & Dobbs, 2017; Pagenkopp Lohan et al., 2016, 2017). Ballast water has long been considered a potential means of introduction for marine parasites (Howard, 1994), and previous studies have reported the presence of some marine parasites and pathogens in ballast water (Aguirre-Macedo et al., 2008; Pagenkopp Lohan et al., 2016, 2017). Moreover, many microscopic parasites have traits that will likely enable them to be successful invaders (Pagenkopp Lohan et al., 2020). Despite the potential of parasites to impact populations and communities upon introduction to new regions, there is yet to be a comprehensive analysis of the diversity of parasites present in ballast water.

In prior studies, we used metabarcoding to identify the diversity of protists (Pagenkopp Lohan et al., 2017) and zooplankton (Darling et al., 2020) in the ballast water (BW) of ships entering three US ports, including a comparison of those ships entering the port of Valdez, AK with and without BW exchange—a treatment method to reduce the transfer of non-native coastal species among regions (Darling et al., 2018). In addition to highlighting the utility of metabarcoding for identifying the diversity of taxa in BW, these studies demonstrated that the technique is sensitive enough to determine changes in community composition after BW exchange (Darling et al., 2018) and the similarities and differences in biodiversity across the BW in ships entering different ports, which is primarily driven by differences in source regions (Darling et al., 2020; Pagenkopp Lohan et al., 2017). Finally, initial examination of taxa showed that all these studies recovered potentially non-native and parasitic taxa in the BW samples examined, leading to our interest in further exploring the richness, taxonomic breadth and geographic distribution of parasitic taxa across these datasets.

In this paper, we combine these previous datasets to more fully evaluate the breadth of marine parasites associated with the BW of ships entering ports across three US coasts. These datasets captured the zooplankton community via plankton tows (Darling et al., 2018) and the protistan community via water samples (Pagenkopp Lohan et al., 2017). All these datasets included samples from ballast tanks of ships entering ports along the Atlantic, Gulf and Pacific US coasts, including Virginia, Texas and Alaska (respectively), with each having vessel traffic (and BW) from different geographic source regions (Miller et al., 2011). Our objectives were to examine the richness and abundance of parasitic taxa (1) associated with all datasets, and (2) across US ports (when applicable) to assess the biosecurity threat posed by shipping regarding potential marine parasite introductions. Our analysis provides the most comprehensive assessment to date of parasites associated with the global transport of ships’ BW.

## 2 | METHODS

Sample collection, processing, library preparation and bioinformatics for all these datasets are described in Darling et al. (2018) for the zooplankton tows and Pagenkopp Lohan et al. (2017) for the ballast water samples. These datasets include (1) the zooplankton community via plankton tows with 80 μm mesh size (Darling et al., 2018) (hereafter referred to as the “80 μm dataset”); (2) the zooplankton community via plankton tows with 35 μm mesh size (Darling et al., 2018) (the “35 μm dataset”); and (3) the protistan community via water samples filtered at 3 μm (Pagenkopp Lohan et al., 2017) (the “3 μm dataset”). In all these datasets, the micro and macroparasites in the zooplankton communities likely include individuals at various life stages. We reiterate this information briefly below.

Because these studies were conducted independently, all sampling methods were not conducted on all ships and the library preparation methods for these datasets also differed. Given these considerations and our focus on parasitic taxa, we assessed diversity only of taxa identified taxonomically as parasites based on comparison of OTUs with existing reference databases, rather than directly assessing total OTU diversity. We recognize the limitations of this approach and discuss them below.

### 2.1 | Sample collection and processing

For this study, we included all the zooplankton samples collected in the larger dataset described in Darling et al. (2018), which are all included in Darling et al. (2020). Additionally, all ballast water samples described in Pagenkopp Lohan et al. (2017) were included. All three datasets contained samples from ballast tanks of ships entering Virginia, Texas and Alaska (hereafter referred to as arrival regions), with 19 ships having samples in all three datasets (for sample info see Table 1). Ballast tanks were accessed via a manhole on deck. For the plankton tows described in Darling et al. (2018), plankton nets with either 80 or 35 μm mesh were lowered separately into the tank until the cod end reached the bottom of the accessible tow depth. The nets were towed vertically through the water column at a consistent speed to the surface of the tank. A manual spray washer was used to rinse the nets and cod ends with filtered tank water, and the sample was collected in a 125-ml Nalgene sample bottle. Zooplankton tow samples were filtered using a 35 μm mesh and preserved in 95% ethanol. Prior to processing, the sample was filtered again using a 20 μm mesh and rinsed with 95% ethanol into a 50-ml Falcon tube. The ballast water (BW) samples described in Pagenkopp Lohan et al. (2017) were obtained using a bleach-washed Kemmerer sampler. Water was dispensed into new sterile or bleach-washed 1-L bottles, which were closed or covered immediately before and after sampling. One litre of BW was filtered using a vacuum pump through a 3 μm, 47 mm nucleopore Whatman filter (VWR International, Atlanta, GA, USA) placed with single-use sterile forceps onto a single-use sterile filter apparatus (Fisher Scientific, Inc., Pittsburgh, PA, USA). Filters were stored at or below −20°C until DNA extraction.

### 2.2 | Amplicon library preparation

The filters resulting from the zooplankton tows were extracted using phenol-chloroform. The BW filters were extracted with the PowerWater DNA Isolation Kit (MoBio Laboratories, Inc., Carlsbad, CA, USA). For both libraries, extraction and PCR-negative controls were carried through the entire library preparation and sequencing. For both libraries, amplicons were generated targeting the small subunit (SSU) gene of the ribosomal RNA (rRNA), although different primers sets were used in the two studies. Thus, the zooplankton tows and BW samples were analysed separately in this study. Subsequent cleaning and library preparation of amplicons for dual-indexing PCR and MiSeq Illumina sequencing were conducted using standard protocols for both datasets (Darling et al., 2018; Pagenkopp Lohan et al., 2017).

### 2.3 | Bioinformatics

For both datasets, OTU tables were generated using similar bio-informatic protocols. Briefly, sequences were merged by sample, and then all low-quality sequences and primers were removed. Sequences were dereplicated, then clustered at 97% similarity. Taxonomy was assigned to each OTU in 3 μm dataset using the RDP classifier v2.2 (Wang et al., 2007) implemented in QIIME (Caporaso et al., 2010) at 70% confidence threshold using the SILVA reference database (Quast et al., 2013). Taxonomy was assigned to each OTU in the 35 and 80 μm datasets using the RDP classifier v2.2 as implemented in QIIME using a custom reference library from sequences collected from the NCBI nt database. For additional details, see (Darling et al., 2018; Pagenkopp Lohan et al., 2017). From each of these OTU tables, parasitic taxa were identified based on taxonomic assignments at the appropriate taxonomic level. For example, we used Rohde (2005) and Roberts and Janovy (2005) to determine which taxonomic rankings contained parasites. If orders or classes are known to only contain parasites, all OTUs identified within those were included. For those orders, families and genera that were noted in Rohde (2005) and Roberts and Janovy (2005) as containing a mix of parasitic and free-living species, we conducted literature searches to find information in the peer-reviewed literature that identified those species as parasitic or not. If the appropriate life history information could not be found, the organism was excluded from our analyses. Subsets of each dataset containing only parasitic taxa were generated and used for further analyses. Given that many parasitic taxa are unlikely to have sequences in reference databases, this method provides a minimum estimate of the total species richness of parasite taxa present in these samples.

### 2.4 | Statistical analyses

All statistical analyses were performed in R (Team, 2020). For all three datasets, the phyloseq package (McMurdie & Holmes, 2013) was used to generate basic statistics, including total number of sequences, samples and OTUs. Venn diagrams were generated using the package VennDiagram (Chen & Boutros, 2011). OTU extrapolation curves were generated using the iNext function with the incidence frequency method and 1000 bootstraps using the iNext package (Hsieh et al., 2016). For the extrapolation curves in iNext, we extrapolated to two times the sample size, which is the maximum recommended in the software documentation. Alpha diversity was calculated in the phyloseq package using the plot_richness function with the observed and Chao 1 diversity metrics. For the remaining analyses that assess relative abundance of taxa, the number of sequences was normalized by dividing the number of sequences per OTU per sample by the total number of OTUs per sample from the original dataset (including non-parasitic taxa). OTUs were then merged within datasets by taxon names at the species level in phyloseq, and heat maps were generated in Microsoft excel. Plots assessing taxonomic richness were generated in phyloseq using the plot_richness function. ANOVAs were conducted using the stats package in R (Team, 2020) on each individual dataset to assess the contribution of richness across the three regions using the Chao1 diversity index. When significance was detected, ad hoc Tukey HSD tests conducted using the agricolae package (de Mendiburu & Yaseen, 2020) in R to determine which regions were significantly different.

To assess taxonomic diversity across the size fractions, all three datasets were merged by taxonomic identification at the order, family and genus levels (hereafter referred to as the “all parasites” dataset). As different primers are known to amplify different taxonomic groups with varying efficiencies, relative abundances would be difficult or impossible to compare across our three datasets. We therefore chose to convert the all parasites dataset to presence/ absence prior to any analyses. For the all parasites dataset, we assessed alpha diversity, then created OTU accumulation curves and venn diagrams across arrival regions and datasets. PERMANOVAs were conducted using the adonis function in the vegan package (Okasanen et al., 2014) using the default parameters on a Bray Curtis distance matrix.

Finally, we used Fastspar (Friedman & Alm, 2012; Watts et al., 2019) across the original entire datasets, containing parasitic and non-parasitic taxa, to test if there were correlations between parasitic and non-parasitic OTUs. We used this approach to explore evidence of association, seeking possible insight as to whether these parasites were infecting holoplanktonic adults in the zooplankton or were free-living, transmissive stages of the parasites detected. For this analysis, the default parameters were used with the exception that the number of iterations was increased to 100. Additionally, for the 3 μm dataset, all OTUs that occurred in 2 or less samples were removed prior to running the analysis.

## 3 | RESULTS

Parasitic taxa ranging from protists to metazoa were identified in all three datasets. In the 80 μm dataset, 98 parasite OTUs were identified in 89% (*n* = 76) of the samples accounting for 22,565 sequences (0.4%; Table 1). Most samples containing parasites (71%, *n* = 54) had <100 sequences from parasitic taxa. In the 35 μm dataset, 115 parasite OTUs were identified in 74% (*n* = 106) of the samples collected accounting for 60,209 sequences (1.1%; Table 1). Again, most of the samples containing parasites (63%, *n* = 67) had <100 sequences from parasitic taxa. In the 3 μm dataset, 783 parasite OTUs were identified in 100% of samples, accounting for 246,114 sequences (11.3%; Table 1), with all but two of the parasitic OTUs identified as protists.

**FIGURE 1:**
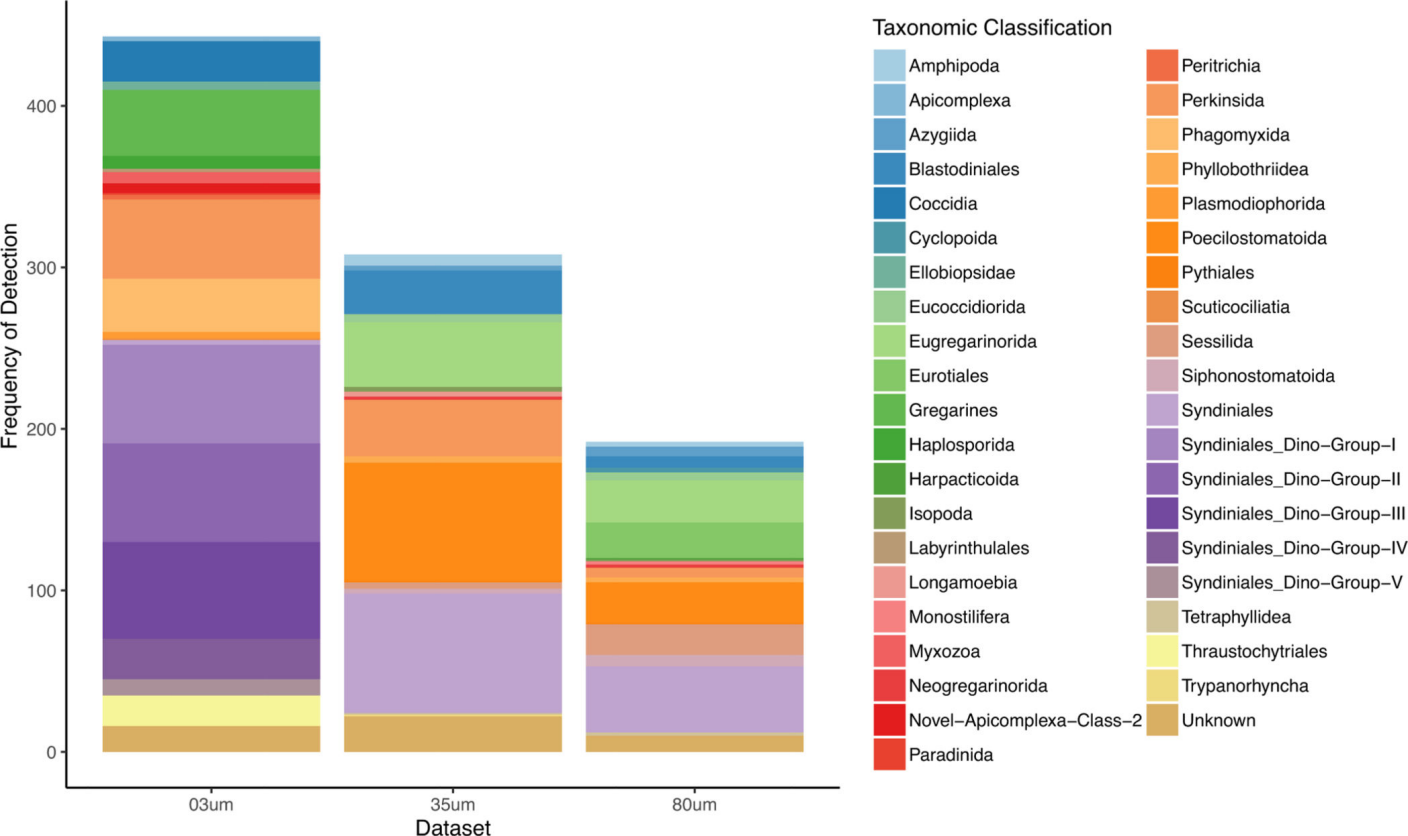
The frequency of detection of OTUs identified at the Order level from the all parasites dataset, with those detections then distributed across the three datasets analysed in this study

After merging all three datasets by taxonomy, 136 parasitic genera, 105 parasitic families and 41 parasitic orders were identified, with the mesh size, region and the interaction between them all being significantly different factors in this dataset (PERMANOVA, Dataset df = 2, sumsq = 27.3, meansq = 13.7, Fmodel = 57.1, R^2^ = 0.3, *p* < .001; Region df = 2, sumsq = 4.1, meansq = 2.06, Fmodel = 8.6, R^2^ = 0.045, *p* < .001; Dataset:Region df = 4, sumsq = 2.7, meansq = 0.7, Fmodel = 2.8, R^2^ = 0.29, *p* < .001). Examining the frequency of detection for each OTU identified by the parasite orders across the all parasite dataset (Figure 1), there is higher detection frequency of certain groups, which varies across the datasets. Parasitic dinoflagellates are found in all datasets, but comprise more of the parasitic taxa found in the 3 μm dataset compared with the other two. In the 35 and 80 μm datasets, most of the parasitic taxa present are copepods (Poecilostomatoida) and ciliates (Sessilida). While there is clear variation in the datasets based on the size fractions sampled, examining the distribution of parasitic orders by arrival region (Figure S1) shows no obvious pattern of parasitic taxa distribution across arrival regions.

**FIGURE 2:**
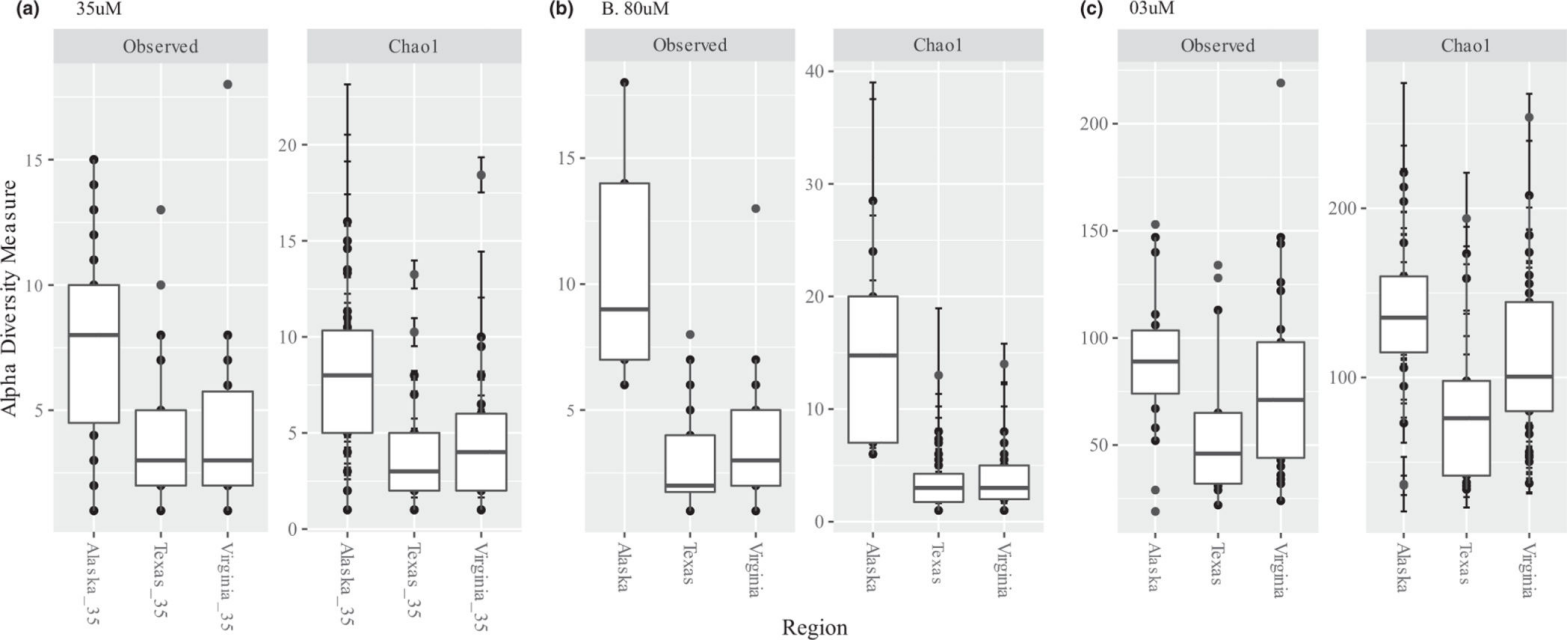
The observed and Chao1 alpha diversity estimates across the three arrival regions within each of the three datasets (a. 35 μm, b. 80 μm and c. 3 μm) examined

Across the three datasets, parasite alpha diversity was highest in the 3 μm and relatively similar in the 35 and 80 μm datasets (Figure 2). ANOVAs run independently on each of the three datasets demonstrated that richness was not significantly different across regions for the 3 μm, but was significantly different for the 35 and 80 μm datasets (Table S1). Post ad hoc Tukey tests showed that the richness of taxa in BW entering Alaska was significantly higher than the richness of taxa in BW entering either Texas or Virginia (Table S1).

Examining the distribution of parasitic taxa across arrival ports, the relative abundance (based on number of sequences) of parasitic taxa in the 35 and 80 μm datasets was quite variable across ports and mesh sizes (Table S2). Sixteen parasitic taxa were represented in the dataset by >500 sequences. While most taxa were in relatively low abundance, a few taxa were in high relative abundance within an arrival port (e.g., *Probopyrus* sp. with 11,978 sequences recovered from ships entering Virginia), while others were found in high abundance across multiple arrival ports (e.g., *Amoebophyra* with >100 sequences across both mesh sizes for all arrival ports). In the normalized heat maps for the 35 and 80 μm datasets, no taxon is the most abundant in all arrival regions or mesh sizes (Table S2). In the 3 μm dataset, most of the parasitic taxa (79%, *n* = 621 OTUs) and 98% of sequences were identified as belonging to the Syndiniales (Table S3). Of the 15 taxa identified as “high abundance” (those with >1000 sequences), 93% (*n* = 14) were identified as belonging to the Syndiniales, while the remaining OTU was identified as belonging to the Perkinsida (Table S2). Examining the 3 μm dataset, the most abundant taxa across arrival regions are the Syndiniales, with syndinids in Dino Group 1-Clade 1 being the most abundant in the BW entering all three arrival regions (Table S2).

**FIGURE 3:**
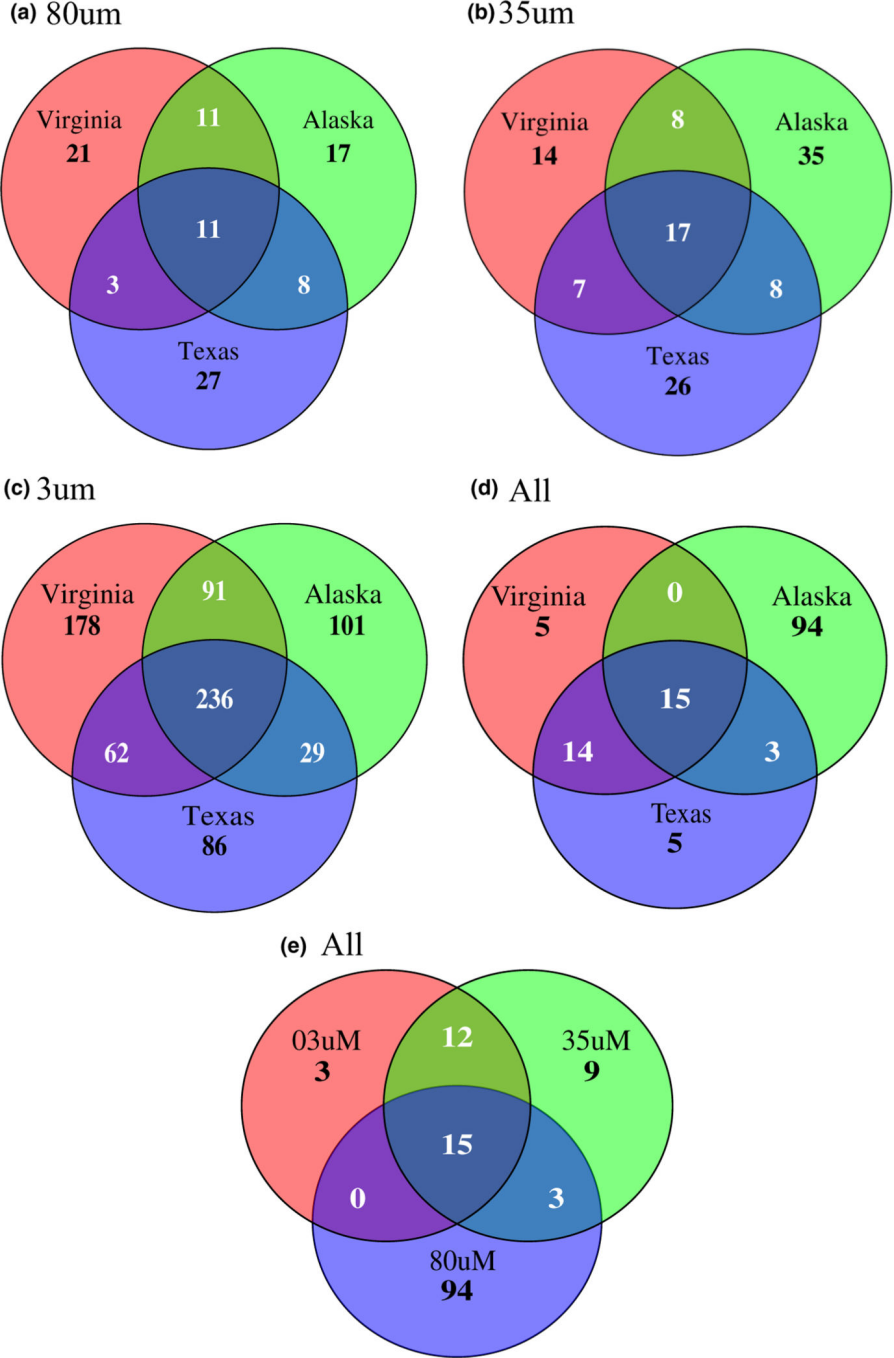
Venn diagrams showing the shared and unique OTUs across regions in each of the datasets examined

For the 35 and 80 μm datasets, we detected minimal overlap (14.7% of 35 μm and 11.4% of the 80 μm datasets) in parasitic OTUs across the three arrival ports, regardless of mesh size, with most OTUs arriving at only a single port (Figure 3a,b). In contrast, in the 3 μm dataset (Figure 3c), 30% (*n* = 236) of parasite OTUs were detected in ships arriving to all three ports, while 46% of OTUs are detected in ships arriving to only a single port. Across the datasets (Figure 3a,b), most of OTUs shared across all three arrival ports were syndinids (5/11 OTUs in the 80 μm dataset; 7/17 OTUs in the 35 μm dataset; 213/236 OTUs in the 3 μm dataset). Examining OTUs shared across multiple arrival regions, most of these taxa were parasitic copepods or arthropods, syndinids, or apicomplexans. When all parasites are merged across the datasets by the genus-level identification (Figure 3d), most parasitic genera were unique to ships entering Alaska. Fifteen parasitic genera were found in ships entering all three regions and across all three datasets (Figure 3d,e). Many of these taxa were identified as parasitic protists, including *Amoebophyra* spp., *Blastodinium* spp., *Hematodinium* spp., *Parvilucifera* spp. and *Syndinium* spp.

**FIGURE 4:**
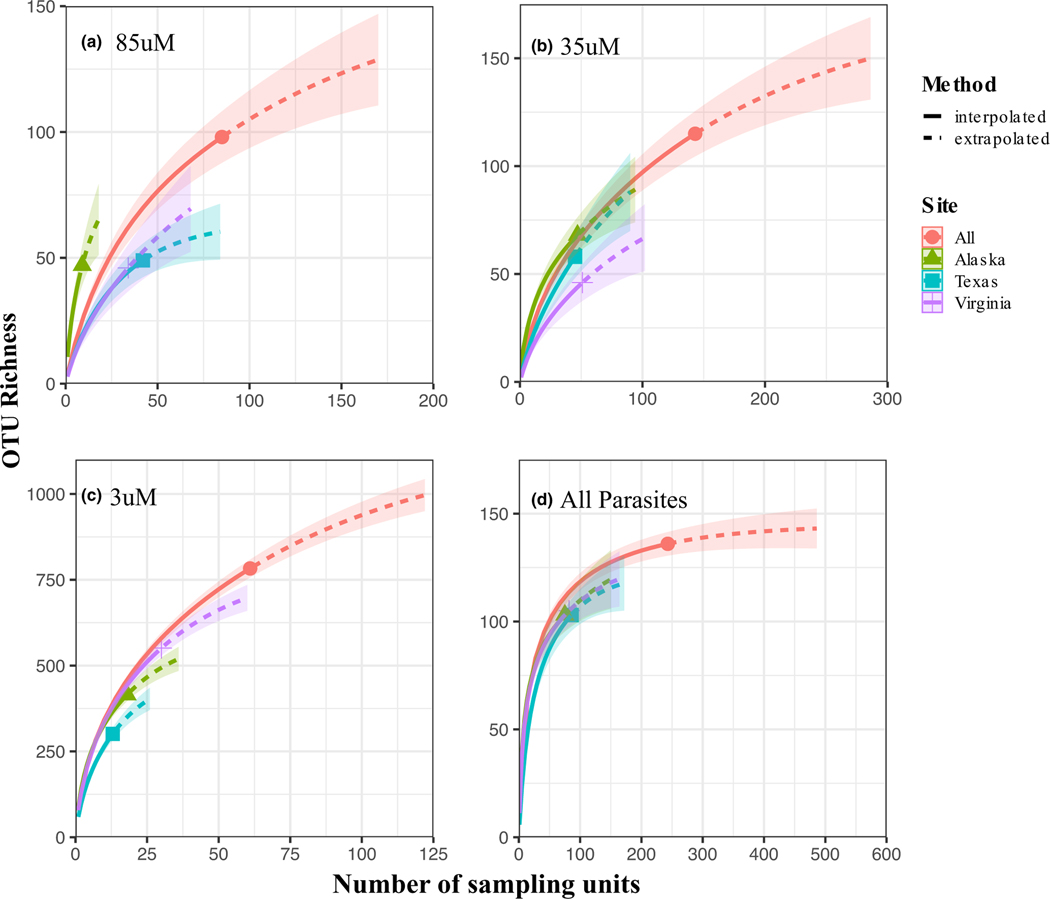
OTU accumulation curves with interpolated (solid lines) and extrapolated (dashed lines) estimates for the 80 (a), 35 (b), 3uM (c), and the all parasites dataset combined based on genera (d) with all arrival regions combined (All), then separately across the arrival regions. Note the differences in the *x* and *y* axes across a–d

For the 85, 35 and 3 μm datasets, only the 85 μm accumulation curve for arrivals into Texas shows the beginning of an asymptote, with an additional ~25 samples needed to reach the asymptote. None of the other accumulation curves across the three datasets showed asymptotes for parasitic taxa, regardless of arrival port (Figure 4). Additionally, extrapolations of those curves indicate that doubling the sample sizes would still be insufficient to cause the curves to asymptote. Examining the all parasites dataset merged at the genus-level, only the accumulation curve for all the data combined appears to begin to asymptote (Figure 4). In contrast, none of the accumulation curves for the arrival regions reach an asymptote, although the extrapolation curves show that doubling the sample size may cause these curves to reach an asymptote. The sample completeness curves with the same data indicate that many additional samples (>100) would be needed to reach 100% of the estimated parasite diversity for the 80 and 35 μm datasets, less additional samples (~50) are needed to reach 100% of the estimate parasite diversity for the 3 μm dataset (Figure S2). Additionally, it appears that ~250 samples are needed to capture 100% of the parasite genera (Figure S2).

Based on the Fastspar results, there were 14 OTUs and 10 OTUs in the 80 μm and 3 μm datasets, respectively, that were significantly correlated (Supporting Information Table S4), while the 35 μm dataset had no significantly correlated OTUs. In the 80 μm dataset, 79% (*n* = 11) of the OTU pairs that were significantly correlated were both identified as parasitic taxa. In the 3 μm dataset, all the pairs of significantly correlated OTUs contained a parasite and non-parasite, which could all be holoplankton, although the taxonomic assignments were not all sufficiently well resolved to verify that status for all the correlated taxa.

## 4 | DISCUSSION

Non-native species are primarily introduced to coastal waters via maritime traffic, either through ballast water discharged by vessels or through biofouling organisms on ships’ hulls (Bailey et al., 2020), which is likely to increase with the expansion of the volume of goods transported by the global shipping fleet. To date, most studies have focussed on the macrofauna present in ballast water, leaving parasites an under-explored component of these potential transfers in ballast water (Pagenkopp Lohan et al., 2020). As parasites have such vital ecological and economic impacts, assessing the risk of transfer via ballast water is critical for the health of coastal ecosystems (Harvell et al., 2004). In this study, we evaluated the breadth of marine parasites associated with the zooplankton and phytoplankton communities in the BW of ships entering ports along the Atlantic, Gulf and Pacific US coasts. These datasets revealed a high diversity of parasites associated with different size fractions of ballast water, ranging from arthropods and cnidarians to protists. Most parasitic taxa identified in the water samples and those most commonly shared across arrival regions belonged to the Syndiniales, which are parasitic dinoflagellates (Coats, 1999). Conversely, several parasites were found at low relative abundances in the 35 and 80 μm datasets, with only a few found in high relative abundance. Additionally, our findings indicate that many of these parasites are found in multiple samples entering one or more than one US port, providing the potential for high propagule pressure for some parasitic taxa. Future research is urgently needed to determine the fate of these parasites after being released with the BW and whether this differs for parasites infecting zooplankton or those free-swimming in the water.

Multiple previous papers have examined the utility and pitfalls of using metabarcode data for examining the diversity of organisms present in ballast water samples and for early detection of invasive species (Brown et al., 2016; Pochon et al., 2017; Zaiko, Martinez, Schmidt-Petersen, et al., 2015), particularly the usefulness of this technique as a management tool (Darling et al., 2017; Darling & Frederick, 2018). Generally, genetic methods, including next-generation sequencing (NGS) combined with metabarcoding, have been proposed as a promising tool for detecting aquatic invasive species because of their high sensitivity, allowing for the detection of cryptic organisms (e.g., larval stages) and low quantities of individuals that may not be easily found in hard to sample or low visibility environments (Darling & Mahon, 2011). Additionally, DNA-based methods can be cheaper and allow for rapid processing of many samples, increasing the likelihood of detection (Darling & Mahon, 2011). On the contrary, there are many technical considerations for adopting molecular approaches for management purposes (Darling & Mahon, 2011). Studies continue to assess the utility of metabarcoding for addressing biosecurity issues related to shipping (Pochon et al., 2017; Zaiko, Martinez, Ardura, et al., 2015) and test the impact of various decisions in the workflow on how these choices might impact analytical outcomes. Most importantly, accurate taxonomic assignments rely on well-populated reference databases based on accurate descriptions of organisms. For parasites, there is an overall lack of knowledge about the total species richness, particularly for viral, bacterial and protistan parasites (Carlson et al., 2020; Dobson et al., 2008). Thus, while metabarcoding may be the best tool currently available for assessing parasite diversity in environmental samples (Bass et al., 2015), due to the dearth of knowledge on parasite diversity on a global scale (Poulin, 2014), this approach may greatly underestimate parasite communities in ballast water and other environmental samples due our inability to assign taxonomy to undescribed parasitic taxa.

To assess the full taxonomic breadth across the identified parasites present in ships’ BW, we took a novel approach to our analyses by merging three independent datasets using the taxonomic information assigned to OTUs. In addition to demonstrating the overlap in diversity across these datasets, we also demonstrate a strategic way the many metabarcode datasets that currently exist, even those generated using different collection and amplification techniques, could potentially be merged to conduct certain explorations of diversity, with necessary caveats. For example, in this study, the mesh size and primer set were confounding factors, so we could not determine whether differences in richness due to mesh size were a result of different taxa captured across mesh sizes or a result of variations in amplification of different taxa by different primer sets. One way to assess this in future studies would be to merge enough datasets to create a large enough set of data to tease apart the various factors influencing alpha and beta diversity metrics. In this way, future studies might further leverage the vast sequence resources available in public databases to examine broadscale diversity patterns. With appropriate care taken in choice of analyses conducted on merged datasets, this could provide further information regarding biogeography and richness of taxa across the globe.

Historically, ships’ BW was not expected to be a major source of invasive parasites, mainly because the primary constituents considered in BW are larval and juvenile stages of hosts, which are less likely to be infected, as parasite prevalence and intensity is often size- and age- dependent (Lafferty & Kuris, 1996; Torchin & Lafferty, 2009). However, results from this study and others (Kim et al., 2015; Pagenkopp Lohan et al., 2016, 2017) suggest that infected adult holoplankton (e.g., copepods, tintinnids and dinoflagellates) are present in BW. For example, *Euduboscquella* spp., which primarily infect tintinnid ciliates (Coats, 1999), have previously been reported in BW (Pierce et al., 1997). While many of the parasites detected in this study are known to infect holoplankton, indicating that infected adult stages could be the culprit, our attempts to provide analytical data to support this notion did not result in a high level of confidence. We attempted to find correlated pairs of OTUs to determine whether this method could identify potential host–parasite associations across BW samples. In the 80 μm dataset, the only significantly correlated OTU pairs had both OTUs identified as parasitic taxa, which could indicate instances of hyperparasitism (when a parasite infects another parasite) or infection of a host that does not exist in the reference database. In the 3 μm dataset, all the OTU pairs of significantly correlated taxa were between a parasite and a free-living holoplanktonic animal, although the lack of resolution in the taxonomic assignments lowers our confidence in this determination. Thus, future studies will be needed to identify the life cycles and potential host range of many of the parasites found. It is unclear if the transmissive or infecting stages of the parasites can better survive the voyage and establish in new areas, as survivability during and after the voyage is likely species-specific and dependent on many factors. Also, critically important to determining establishment risk is the availability of competent hosts in the recipient environment. Given that species transported by BW may be exposed to entirely novel environments compared with native ranges, the ability of BW-borne parasites to successfully infect and achieve population-level transmission is not guaranteed and will depend on both the composition of recipient biota, host specificity and even the capacity of introduced parasites to adapt to novel hosts (Tepolt et al., 2020).

While the observed disparity in parasite richness across datasets (Figure 2) could be due to technical differences across the datasets, the similarity in the composition of organisms within BW entering the same arrival region is generally driven by similarity in the source region (i.e., where the ship and its BW came from), due to global shipping patterns (Miller et al., 2011). We suspect the disparity in parasite richness between the water (3 μm) and zooplankton (35 μm and 80 μm) datasets could be due to (1) the variation in abundance of parasites across size groups within the water (i.e., more free-living stages of parasites present and thus detected in small size classes vs. infected adults) or (2) the different primer sets varied in their ability to amplify the parasitic taxa present. Future direct comparisons across datasets with the same primer set could determine the taxonomic differences across sample types. Interestingly, 15 parasitic genera were found in ships entering all three regions and across all three datasets, most of which were parasitic protists, including syndinids. Additionally, parasitic copepods and syndinids were most likely to appear in BW across regions in the 35 and 80 μm datasets, while syndinids were most likely to appear in BW across regions in the 3 μm dataset. In fact, the parasitic dinoflagellates (i.e., syndinids) were found in all three datasets, while comprising most of the parasitic taxa found in the 3 μm dataset. The parasitic taxa in the 35 and 80 μm datasets were primarily parasitic copepods (Poecilostomatoida) and ciliates (Sessilida).

We previously identified several traits that we hypothesized would increase the likelihood of parasites surviving and establishing in a new geographic region as a non-native species (Pagenkopp Lohan et al., 2020), in addition to the need for competent host(s) in the arrival region. These traits included a direct life cycle, low host specificity, a long-lived transmission stage, facultative parasitism and a resistant or dormant stage. The results of this study indicate there are likely many parasites that are at least host generalists with direct life cycles in the BW of many ships entering US waters. Identified across all datasets and in high abundance across arrival regions, the Syndiniales are obligate, parasitic dinoflagellates that infect a wide range of host taxa, from crustaceans such as copepods to other dinoflagellates, with their life cycles only requiring a single host (Coats, 1999). Parasites of copepods (e.g., *Zoothamnium* spp. and *Syndinium* spp.) and dinoflagellates (e.g., *Amoebophyra* spp., *Blastodinium* spp. and *Syndinium*) were identified, including some in high relative abundance (*Zoothamnium* spp. and *Amoebophyra* spp.) (Figure 1). Many of these genera are known to infect a wide range of hosts (Coats, 1999). Additionally, we found parasitic copepods from two orders and five families, including at least eight genera (Figure 3). Some of these genera, including those in the family Caligidae, generally have direct life cycles and broad host ranges and have a propensity for being problematic in marine aquaculture (Johnson et al., 2004). Thus, our data contribute to the growing body of literature demonstrating that ballast water is a potent vector for a wide range of marine parasites, including many taxa that have life history traits amenable to surviving transport and establishing in a new geographic area where they could infect and disrupt naïve populations and communities.

The current study highlights the extent of parasite transfers associated with BW that have been occurring and expanding on a global scale for the last century. Although the most comprehensive such analysis to date, this is still a relatively coarse measure that detects only a fraction of the diversity of parasites present in BW, due to current technical constraints and taxonomic resolution. We surmise that there are substantial opportunities for parasites, and other microbes, to invade new areas due to BW and other vectors. While invasions of parasites are known (e.g., Lymbery et al., 2014; Torchin & Mitchell, 2004), identifying the mechanism for introduction is often difficult, as there are multiple natural and anthropogenic vectors capable of spreading parasites across marine habitats (Pagenkopp Lohan et al., 2020). While multiple studies have suspected that shipping likely played a role in the spread of marine parasites (Bishop et al., 2006; Burreson & Ford, 2004; Howard, 1994; Pagenkopp Lohan et al., 2018), no study has been able to more definitively determine that ships were the sole vector for introducing a parasite to a new coastal area. Thus, it is imperative that parasites and other microbes receive more focus in invasions studies, as they could have out-sized impacts after establishment in new areas.

## 5 | FUTURE CONSIDERATIONS

In September 2017, the International Convention for the Control and Management of Ship’s Ballast Water and Sediment, more commonly referred to as the BWM Convention, went into effect. This agreement includes regulations for on-board ballast water treatment technologies to reduce the number of organisms that are present in discharged ballast water (Darling & Frederick, 2018). Unfortunately, there is no general numerical discharge standard for organisms <10 μm, which encompasses most parasites found in this study. Rather, there are specific guidelines for identifying three indicator taxa: (1) toxigenic *Vibrio cholerae* (serotypes 01–0139) must be at a concentration of less than 1 colony-forming unit (CFU) per 100 ml, (2) *Escherichia coli* must be at a concentration of less than 250 CFU per 100 ml, and (3) intestinal enterococci must be at a concentration of less than 100 CFU per 100 ml. It is highly unlikely that three bacterial taxa are sufficient or robust indicators for the broad diversity of protistan and metazoan parasites also found in BW, such as in this study. Critically, the testing for the treatment technologies did not assess their effectiveness at removing many parasites <10 microns in BW, as there are no discharge standards for organisms other than those indicator taxa. The use of only human pathogens as indicator species negates the potential for major ecological and economic impacts associated with the invasion of marine parasites of plants and animals (Lafferty, 2017).

## 6 | CONCLUSION

Given the increase in global shipping and in outbreaks of marine disease globally, additional research is urgently needed to examine the fate of these parasites in BW. Our study demonstrates that there is a high diversity of parasitic taxa present in BW, that some of these parasitic taxa are likely to be host generalists, and others are likely infecting adult holoplankton. To better understand the potential impacts of these parasites in coastal waters, future research is needed to examine (1) how parasites fare after exposure to certain treatment technologies, (2) the likelihood of these different taxa to survive transit, and, perhaps most importantly (3) the likelihood that these different taxa can establish in new areas. These additional data are critical for guiding future management decisions and preventing future marine disease outbreaks.

## Supplementary Material

Supplement1

## Figures and Tables

**TABLE 1:** The number of ships where BW was sampled, number of BW samples collected and number of sequences obtained (after quality control) from the entirety of all three datasets

Dataset	Total ships	Total samples	Total sequences	Samples with parasites	Parasite sequences	Parasite OTUs
3 μm	39	61	2,176,892	61	246,114	783
35 μm	143	143	5,669,876	106	60,209	115
80 μm	85	85	5,138,836	76	22,565	98

*Note*: Additionally, the number of samples, sequences and OTUs that were identified as parasites from all three datasets are shown.

## Data Availability

Raw sequence files were deposited in GenBank under NCBI BioProjects PRJNA371431 and PRJNA649942.

## References

[R1] Aguirre-Macedo ML, Vidal-Martinez VM, Herrera-Silveira JA, Valdes-Lozano DS, Herrera-Rodriguez M, & Olvera-Novoa MA (2008). Ballast water as a vector of coral pathogens in the Gulf of Mexico: The case of the Cayo Arcas coral reef. Marine Pollution Bulletin, 56(9), 1570–1577. 10.1016/j.marpolbul.2008.05.022 18639903

[R2] Bailey SA, Brown L, Campbell ML, Canning-Clode J, Carlton JT, Castro N, Chainho P, Chan FT, Creed JC, Curd A, Darling J, Fofonoff P, Galil BS, Hewitt CL, Inglis GJ, Keith I, Mandrak NE., Marchin A., McKenzi CH., … Husse N. (2020). Trends in the detection of aquatic non-indigenous species across global marine, estuarine and freshwater ecosystems: A 50-year perspective. Diversity and Distributions, 26(12), 1780–1797. 10.1111/ddi.13167 36960319 PMC10031752

[R3] Bass D, Stentiford GD, Littlewood DTJ, & Hartikainen H. (2015). Diverse applications of environmental DNA methods in parasitology. Trends in Parasitology, 31(10), 499–513. 10.1016/j.pt.2015.06.013 26433253

[R4] Bishop MJ, Carnegie RB, Stokes NA, Peterson CH, & Burreson EM (2006). Complications of a non-native oyster introduction: Facilitation of a local parasite. Marine Ecology Progress Series, 325, 145–152.

[R5] Blakeslee AM, Fowler AE, & Keogh CL (2013). Marine invasions and parasite escape: Updates and new perspectives. Advances in Marine Biology, 66, 87–169. 10.1016/B978-0-12-408096-6.00002-X 24182900

[R6] Brown EA, Chain FJJ, Zhan A, MacIsaac HJ, Cristescu ME, & Lowe A. (2016). Early detection of aquatic invaders using metabarcoding reveals a high number of non-indigenous species in Canadian ports. Diversity and Distributions, 22(10), 1045–1059. 10.1111/ddi.12465

[R7] Burreson EM, & Ford SE (2004). A review of recent information on the Haplosporidia, with special reference to *Haplosporidium* nelsoni (MSX disease). Aquatic Living Resources, 17(4), 499–517. 10.1051/alr:2004056

[R8] Caporaso JG, Kuczynski J, Stombaugh J, Bittinger K, Bushman FD, Costello EK, Fierer N, Gonzalez Peña A, Goodrich JK, Huttley GA, Kelley ST, Knights D, Koenig JE, Ley RE, Lozupone CA, McDonald D, Muegge BD, Pirrung M, … Knight R. (2010). QIIME allows analysis of high throughput community sequencing data. Nature Methods, 7(5), 335–336. 10.1038/nmeth0510-335 20383131 PMC3156573

[R9] Carlson CJ, Dallas TA, Alexander LW, Phelan AL, & Phillips AJ (2020). What would it take to describe the global diversity of parasites? Proceedings of the Royal Society B, 287(1939), 20201841. 10.1098/rspb.2020.1841 PMC7739500 33203333

[R10] Chen H, & Boutros PC (2011). VennDiagram: A package for the generation of highly customizable Venn and Euler diagrams in R. BMC Bioinformatics, 12(35), 35. 21269502 10.1186/1471-2105-12-35 PMC3041657

[R11] Coats DW (1999). Parasitic lifestyles of marine dinoflagellates. Journal of Eukaryotic Microbiology, 46(4), 402–409.

[R12] Darling JA, & Frederick RM (2018). Nucleic acids-based tools for ballast water surveillance, monitoring, and research. Journal of Sea Research, 133, 43–52. 10.1016/j.seares.2017.02.005 30147432 PMC6104837

[R13] Darling JA, Galil BS, Carvalho GR, Rius M, Viard F, & Piraino S. (2017). Recommendations for developing and applying genetic tools to assess and manage biological invasions in marine ecosystems. Marine Policy, 85, 56–64. 10.1016/j.marpol.2017.08.014 29681680 PMC5909192

[R14] Darling JA, & Mahon AR (2011). From molecules to management: Adopting DNA-based methods for monitoring biological invasions in aquatic environments. Environmental Research, 111(7), 978–988. 10.1016/j.envres.2011.02.001 21353670

[R15] Darling JA, Martinson J, Gong Y, Okum S, Pilgrim E, Lohan KMP, Carney KJ, & Ruiz GM (2018). Ballast water exchange and invasion risk posed by intracoastal vessel traffic: An evaluation using high throughput sequencing. Environmental Science & Technology, 52(17), 9926–9936. 10.1021/acs.est.8b02108 30059206 PMC6944436

[R16] Darling JA, Martinson J, Pagenkopp-Lohan K, Carney KJ, Pilgrim E, Banerji A, Holzer KK, & Ruiz GM (2020). Metabarcoding quantifies differences in accumulation of ballast water borne biodiversity among three port systems in the United States. Science of The Total Environment, 749, 141456. 10.1016/j.scitotenv.2020.141456 PMC8190815 32846346

[R17] de Mendiburu F, & Yaseen M. (2020). agricolae: Statistical procedures for agricultural research version 1.4. https://myaseen208.github.io/agricolae/https://cran.r-project.org/package=agricolae

[R18] Dobson A, Lafferty KD, Kuris AM, Hechinger RF, & Jetz W. (2008). Colloquium paper: Homage to Linnaeus: How many parasites? How many hosts? Proceedings of the National Academy of Sciences of the United States of America, 105(Suppl. 1), 11482–11489. 10.1073/pnas.0803232105 18695218 PMC2556407

[R19] Drake LA, Choi KH, Ruiz GM, & Dobbs FC (2001). Global redistribution of bacterioplankton and virioplankton communities. Biological Invasions, 3, 193–199.

[R20] Drake LA, Doblin MA, & Dobbs FC (2007). Potential microbial bioinvasions via ships’ ballast water, sediment, and biofilm. Marine Pollution Bulletin, 55(7–9), 333–341. 10.1016/j.marpolbul.2006.11.007 17215010

[R21] Dunn AM, & Hatcher MJ (2015). Parasites and biological invasions: Parallels, interactions, and control. Trends in Parasitology, 31(5), 189–199. 10.1016/j.pt.2014.12.003 25613560

[R22] Friedman J, & Alm EJ (2012). Inferring correlation networks from genomic survey data. PLoS Computational Biology, 8(9), e1002687. 10.1371/journal.pcbi.1002687 PMC3447976 23028285

[R23] Harvell C, Aronson RA, Baron N, Connell J, Dobson A, Ellner S, Gerber L, Kim K, Kuris A, McCallum H, Lafferty K, McKay B, Porter J, Pascual M, Smith G, Sutherland K, & Ward J. (2004). The rising tide of ocean diseases: Unsolved problems and research priorities. Frontiers in Ecology and the Environment, 2(7), 375–382.

[R24] Hatcher MJ, Dick JT, & Dunn AM (2006). How parasites affect interactions between competitors and predators. Ecology Letters, 9(11), 1253–1271. 10.1111/j.1461-0248.2006.00964.x 17040328

[R25] Hewson I, Button JB, Gudenkauf BM, Miner B, Newton AL, Gaydos JK, Wynne J, Groves CL, Hendler G, Murray M, Fradkin S, Breitbart M, Fahsbender E, Lafferty KD, Kilpatrick AM, Miner CM, Raimondi P, Lahner L, Friedman CS, … Harvell CD (2014). Densovirus associated with sea-star wasting disease and mass mortality. Proceedings of the National Academy of Sciences of the United States of America, 111(48), 17278–17283. 10.1073/pnas.1416625111 25404293 PMC4260605

[R26] Howard AE (1994). The possibility of long distance transmission of *Bonamia* by fouling on boat hulls. Bulletin of European Association of Fish Pathology, 14(16), 211.

[R27] Hsieh TC, Ma KH, Chao A, & McInerny G. (2016). iNEXT: An R package for rarefaction and extrapolation of species diversity (Hill numbers). Methods in Ecology and Evolution, 7(12), 1451–1456. 10.1111/2041-210x.12613

[R28] Johnson SC, Treasurer JW, Bravo S, Nagasawa K, & Kabata Z. (2004). A review of the impact of parasitic copepods on marine aquaculture. Zoological Studies, 43(2), 229–243.

[R29] Kim Y, Aw TG, Teal TK, & Rose JB (2015). Metagenomic investigation of viral communities in ballast water. Environmental Science & Technology, 49(14), 8396–8407. 10.1021/acs.est.5b01633 26107908

[R30] Lafferty K. (2017). Marine infectious disease ecology. Annual Review of Ecology, Evolution, and Systematics, 48, 473–496. 10.1146/annurev-ecolsys-

[R31] Lafferty KD, Dobson AP, & Kuris AM (2006). Parasites dominate food web links. Proceedings of the National Academy of Sciences of the United States of America, 103(30), 11211–11216. 10.1073/pnas.0604755103 16844774 PMC1544067

[R32] Lafferty KD, & Kuris AM (1996). Biological control of marine pests. Ecology, 77(7), 1989–2000.

[R33] Lymbery AJ., Morine M., Kanani HG., Beatty SJ., & Morgan DL. (2014). Co-invaders: The effects of alien parasites on native hosts. International Journal for Parasitology: Parasites and Wildlife, 3(2), 171–177. 10.1016/j.ijppaw.2014.04.002 25180161 PMC4145144

[R34] Lymperopoulou DS, & Dobbs FC (2017). Bacterial diversity in ships’ ballast water, ballast-water exchange, and implications for ship-mediated dispersal of microorganisms. Environmental Science & Technology, 51(4), 1962–1972. 28135081 10.1021/acs.est.6b03108

[R35] McMurdie PJ, & Holmes S. (2013). phyloseq: An R package for reproducible interactive analysis and graphics of microbiome census data. PLoS One, 8, e61217. 10.1371/journal.pone.0061217 PMC3632530 23630581

[R36] Miller AW, Minton MS, & Ruiz GM (2011). Geographic limitations and regional differences in ships’ ballast water management to reduce marine invasions in the contiguous United States. Bioscience, 61(11), 880–887. 10.1525/bio.2011.61.11.7

[R37] Okasanen J, Guillaume Blanchet F, Kindt R, Legendre P, Minchin PR, O’Hara RB, Simpson GL, Solymos P, Stevens M, & Wagner H. (2014). Package “vegan”: Community ecology package. http://cran.r-project.org, http://vegan.r-forge.r-project.org/

[R38] Pagenkopp Lohan KM, Fleischer RC, Carney KJ, Holzer KK, & Ruiz GM (2016). Amplicon-based pyrosequencing reveals high diversity of protistan parasites in ships’ ballast water: Implications for biogeography and infectious diseases. Microbial Ecology, 71(3), 530–542. 10.1007/s00248-015-0684-6 26476551

[R39] Pagenkopp Lohan KM, Fleischer RC, Carney KJ, Holzer KK, Ruiz GM, & Zhan A. (2017). Molecular characterisation of protistan species and communities in ships’ ballast water across three U.S. coasts. Diversity and Distributions, 23(6), 680–691. 10.1111/ddi.12550

[R40] Pagenkopp Lohan KM, Hill-Spanik KM, Torchin ME, Fleischer RC, Carnegie RB, Reece KS, & Ruiz GM (2018). Phylogeography and connectivity of molluscan parasites: *Perkinsus* spp. Panama and beyond. International Journal for Parasitology, 48(2), 135–144. 10.1016/j.ijpara.2017.08.014 29108906

[R41] Pagenkopp Lohan KM, Ruiz GM, & Torchin ME (2020). Invasions can drive marine disease dynamics. In Behringer D, Lafferty K, & Silliman BR (Eds.), Marine disease ecology (pp. 115–138). Oxford University Press.

[R42] Pierce RW, Carlton JM, Carlton DA, & Geller J. (1997). Ballast water as a vector for tintinnid transport. Marine Ecology Progress Series, 149, 295–297.

[R43] Pochon X, Zaiko A, Fletcher LM, Laroche O, & Wood SA (2017). Wanted dead or alive? Using metabarcoding of environmental DNA and RNA to distinguish living assemblages for biosecurity applications. PLoS One, 12(11), e0187636. 10.1371/journal.pone.0187636 PMC5667844 29095959

[R44] Poulin R. (2014). Parasite biodiversity revisited: Frontiers and constraints. International Journal for Parasitology, 44(9), 581–589. 10.1016/j.ijpara.2014.02.003 24607559

[R45] Poulin R. (2017). Invasion ecology meets parasitology: Advances and challenges. International Journal for Parasitology: Parasites and Wildlife, 6(3), 361–363. 10.1016/j.ijppaw.2017.03.006 30951572 PMC5715220

[R46] Quast C, Pruesse E, Yilmaz P, Gerken J, Schweer T, Yarza P, Peplies J, & Glockner FO (2013). The SILVA ribosomal RNA gene database project: Improved data processing and web- based tools. Nucleic Acids Research, 41(Database issue), D590–D596. 10.1093/nar/gks1219 23193283 PMC3531112

[R47] Roberts LS, & Janovy J Jr. (2005). Foundations of parasitology (7th ed.). The McGraw-Hill Companies.

[R48] Rohde K. (2005). Marine parasitology. CSIRO Publishing.

[R49] Ruiz GM, Fofonoff PW, Steves BP, & Carlton JT (2015). Invasion history and vector dynamics in coastal marine ecosystems: A North American perspective. Aquatic Ecosystem Health & Management, 18(3), 299–311. 10.1080/14634988.2015.1027534

[R50] Ruiz GM, Rawlings TK, Dobbs FC, Drake LA, Mullady T, Huq A, & Colwell RR (2000). Global spread of microorganisms by ships. Nature, 408, 49–50. 11081499 10.1038/35040695

[R51] Team RC. (2020). R: Foundation for statistical computing. http://www.r-project.org/

[R52] Tepolt CK, Darling JA, Blakeslee AMH, Fowler AE, Torchin ME, Miller AW, & Ruiz GM (2020). Recent introductions reveal differential susceptibility to parasitism across an evolutionary mosaic. Evolutionary Applications, 13(3), 545–558. 10.1111/eva.12865 32431735 PMC7045710

[R53] Torchin ME, & Lafferty KD (2009). Escape from parasites. In Rilov G, & Crooks JA (Eds.), Biological invasions in marine ecosystems (pp. 203–214). Springer.

[R54] Torchin ME, Lafferty K, & Kuris A. (2002). Parasites and marine invasions. Parasitology, 124, S137–S151. 12396221 10.1017/s0031182002001506

[R55] Torchin ME, Lafferty KD, Dobson AP, McKenzie VJ, & Kuris AM (2003). Introduced species and their missing parasites. Nature, 421(6923), 628–630. 10.1038/nature01346 12571595

[R56] Torchin ME, & Mitchell CE (2004). Parasites, pathogens, and invasions by plants and animals. Frontiers in Ecology and the Environment, 2(4), 183–190.

[R57] Tracy AM, Pielmeier ML, Yoshioka RM, Heron SF, & Harvell CD (2019). Increases and decreases in marine disease reports in an era of global change. Proceedings of the Biological Sciences, 286(1912), 20191718. 10.1098/rspb.2019.1718 PMC6790777 31594507

[R58] Wang Q, Garrity GM, Tiedje JM, & Cole JR (2007). Naive Bayesian classifier for rapid assignment of rRNA sequences into the new bacterial taxonomy. Applied and Environmental Microbiology, 73(16), 5261–5267. 10.1128/AEM.00062-07 17586664 PMC1950982

[R59] Ward JR, & Lafferty KD (2004). The elusive baseline of marine disease: Are diseases in ocean ecosystems increasing? PLoS Biology, 2(4), E120. 10.1371/journal.pbio.0020120 15094816 PMC387283

[R60] Watts SC., Ritchie SC., Inouye M., & Holt KE. (2019). FastSpar: Rapid and scalable correlation estimation for compositional data. Bioinformatics, 35(6), 1064–1066. 10.1093/bioinformatics/bty734 30169561 PMC6419895

[R61] Zaiko A, Martinez JL, Ardura A, Clusa L, Borrell YJ, Samuiloviene A, Roca A, & Garcia-Vazquez E. (2015). Detecting nuisance species using NGST: Methodology shortcomings and possible application in ballast water monitoring. Marine Environmental Research, 112(Pt B), 64–72. 10.1016/j.marenvres.2015.07.002 26174116

[R62] Zaiko A, Martinez JL, Schmidt-Petersen J, Ribicic D, Samuiloviene A, & Garcia-Vazquez E. (2015). Metabarcoding approach for the ballast water surveillance—An advantageous solution or an awkward challenge? Marine Pollution Bulletin, 92(1–2), 25–34. 10.1016/j.marpolbul.2015.01.008 25627196

