# Supplementary material for: International shipping as a potent vector for spreading marine parasites
Source: Divers Distrib. Author manuscript; Available in PMC 2024 Jan 24. (PMC10807284; doi:10.1111/ddi.13592)
Supplement: Supplement1 [file NIHMS1856260-supplement-Supplement1.docx]

Table S1. Results of the ANOVA tests for each dataset using the Chao1 diversity index. The results of the post hoc Tukey HSD test are provided for the two datasets that were significant.

|  | **Degrees of Freedom** | **Sum Squares** | **Mean Square** | **F value** | **P-value** | **Tukey HSD results** |
| --- | --- | --- | --- | --- | --- | --- |
| 03𝜇m | 2 | 336 | 168.2 | 168.2 | 0.923 | NA |
| 35𝜇m | 2 | 359.1 | 179.56 | 13.34 | <0.0001 | Alaska |
| 80𝜇m | 2 | 989.9 | 494.9 | 34.84 | <0.0001 | Alaska |

Table S2. A heat map showing the distribution of the taxa designated as being of high relative abundance (based on number of sequences) across arrival regions in the 35 and 80𝜇m datasets. Taxonomic names for each row are to genus or the lowest level identified (last column). The abundance values are calculated as the proportion of sequences within the column.

Table S3. A heat map showing the distribution of the taxa designated as being of high relative abundance (based on number of sequences) across arrival regions in the 3𝜇m dataset. Taxonomic names are to genus or the lowest level identified. The abundance values are calculated as the proportion sequences within the column.

Table S4. The significantly correlated OTUs identified by FastSpar (Friedman & Alm, 2012; Watts, Ritchie, Inouye, & Holt, 2019) in the 80 and 3𝜇m datasets.


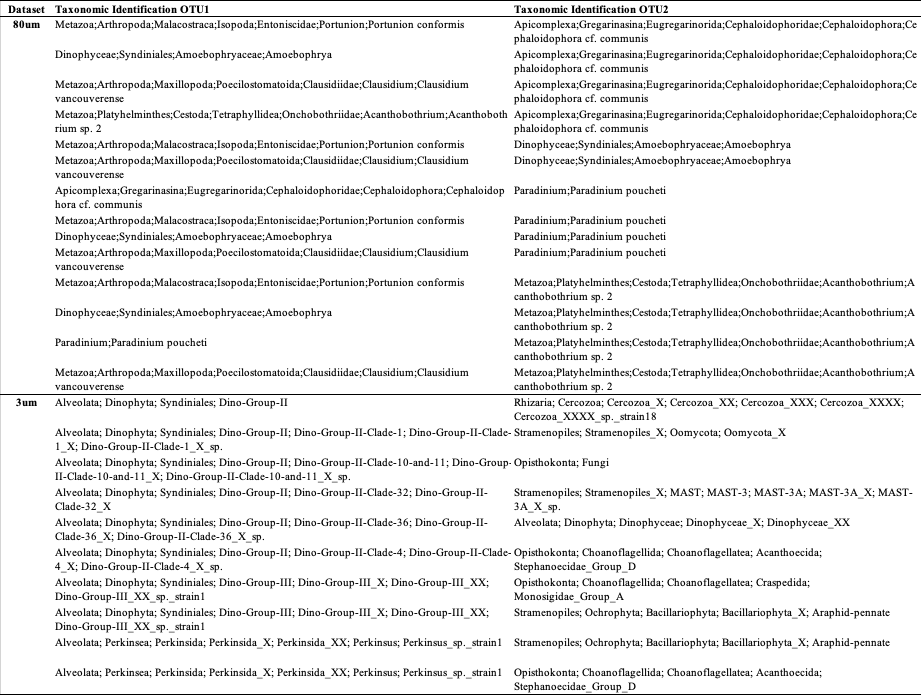


Figure S1. The distribution of parasitic Orders from the all parasites dataset distributed across the three arrival regions analyzed in this study.


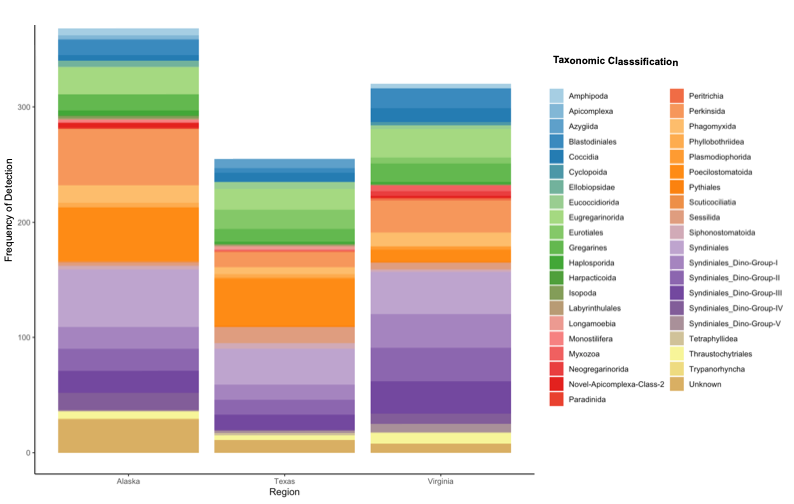


**Figure S2.** Sample coverage curves with interpolated (solid lines) and extrapolated (dashed lines) estimates for the 80 𝜇M (A), 35 𝜇M (B), 3𝜇M (C), and the all parasites dataset combined based on genera (D) with all arrival regions combined (All), then separately across the arrival regions. Note the differences in the x axes across A-D.

**
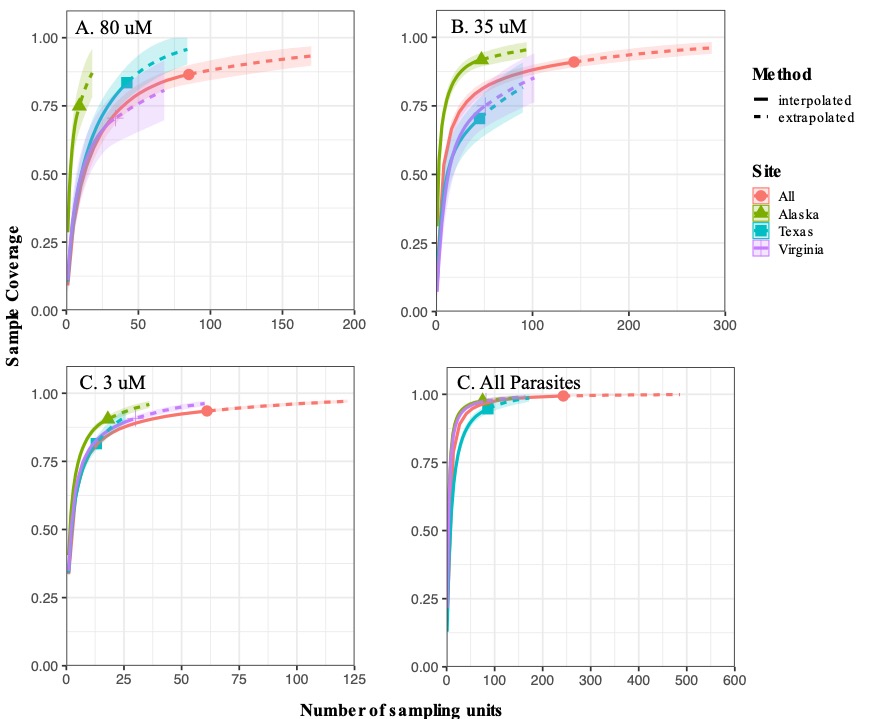
**

**References**

Friedman, J., & Alm, E. J. (2012). Inferring correlation networks from genomic survey data. *PLoS Comput Biol, 8*(9), e1002687. doi:10.1371/journal.pcbi.1002687

Watts, S. C., Ritchie, S. C., Inouye, M., & Holt, K. E. (2019). FastSpar: rapid and scalable correlation estimation for compositional data. *Bioinformatics, 35*(6), 1064-1066. doi:10.1093/bioinformatics/bty734
